# Understanding the interplay between text quality, writing self-efficacy and writing anxiety in learners with and without migration background

**DOI:** 10.3389/fpsyg.2023.1130149

**Published:** 2023-05-23

**Authors:** Vera Busse, Steve Graham, Nora Müller, Till Utesch

**Affiliations:** ^1^Institute of Education, Faculty of Education and Social Science, University of Münster, Münster, North Rhine-Westphalia, Germany; ^2^Division of Leadership and Innovation, Arizona State University, Tempe, AZ, United States

**Keywords:** writing, self-efficacy, anxiety, migration background, student beliefs and values, L2 learners, response surface analyses

## Abstract

Writing presents considerable challenges to students’ motivation. Yet there is a dearth of studies assessing the role of affect and motivation in writing performance for students with migration backgrounds (MB), who often underachieve in writing. Our study addressed this research gap by investigating the interplay between writing self-efficacy, writing anxiety, and text quality in 208 secondary students with and without MB using Response Surface Analyses. The data showed comparable levels of self-efficacy and, notably, lower writing anxiety levels among students with MB despite lower writing achievements. In the full sample, we observed positive correlations between self-efficacy and text quality and negative correlations between writing anxiety and text quality. When modeling efficacy and anxiety measures and their interplay to predict text quality, self-efficacy measures continued to account for statistically detectable unique variance in text quality, whereas writing anxiety did not. However, students with MB demonstrated differing interplay patterns, with less efficacious students with MB showing positive relations between writing anxiety and text quality.

## 1. Introduction

Writing is key for participating in political and societal discourses (Peltzer et al., 2022), succeeding in school (Graham and Perin, 2007), or finding adequate employment in the work sector (National Commission on Writing, 2004; Cellier et al., 2007; Aschliman, 2016). Yet, many teenagers struggle with writing comprehensive texts and fail to reach a satisfactory level of writing proficiency (e.g., National Commission on Writing, 2004). Writing can be particularly demanding for students with migration backgrounds (MB), some of whom may be writing in a second language (L2), which is arguably “one of the most challenging aspects of second language learning” (Hyland, 2003, p. xiii).

Students with MB, albeit by no means a homogenous group, often share a familial history of a migration experience, are more likely to be affected by poverty (OECD, 2010), and generally experience less favorable conditions for language development in the language of school instruction, particularly when that language is not spoken at home (Cummins, 2000; Kempert et al., 2016). In the United States, the National Commission on Writing warned that unless more attention is paid to writing development, students from minority groups and English-language learners may be confined to “low-skill, low-wage, hourly employment” (National Commission on Writing, 2004, p. 19). This warning should also be taken seriously in European societies. Although writing attainment is not measured in large-scale assessments such as PISA, comparatively lower educational attainment in literacy of students with MB compared to students without MB have been amply documented in European countries (Baumert et al., 2006; Stanat and Christensen, 2006; OECD, 2010; Marx and Stanat, 2012; Ohinata and van Ours, 2012). Furthermore, studies focusing on writing reveal achievement disparities between students with and without MB and between first language (L1) and L2 writers (Silva, 1993; Neumann and Lehmann, 2008; Babayiğit, 2015).

Students’ struggles with writing may not only be related to the cognitive challenges posed by writing *per se* but also to ensuing affective- and motivational states and lack of confidence (Bruning and Kauffman, 2016). Research suggests that minority learners often suffer from low writing self-efficacy beliefs or even writing apprehension (Pajares, 1996, 2003). Yet research exploring the relationship between affective-motivational aspects of writing and writing performance is scarce (Camacho et al., 2021), and researchers have paid little attention to students at-risk in writing, including students with MB. Addressing this critical gap in the literature, our study was designed to examine patterns of interplay between text quality, writing self-efficacy, and writing anxiety in students with and without MB. We draw on data from a larger feedback intervention project revealing that secondary students experience difficulties with establishing deep-level features in writing, such as text structure and coherence (Siekmann et al., 2022; Müller and Busse, 2023). In the current study, we were particularly interested in investigating the associated role of writing self-efficacy and anxiety as possible antecedents of writing performance prior to the intervention.

## 2. Theoretical background

### 2.1. Motivation, writing self-efficacy, and writing anxiety

Although the multidimensional concept of motivation has been defined in different ways, most motivational research concerns the direction and magnitude of (learning) behavior. Thereby addressing not only the question of why students choose to do something but also how long they persist and how much effort they expand on the activity (Dörnyei and Ushioda, 2011). Writing is a complex and cognitively demanding activity with high demands on working memory (Hayes and Flower, 1986; see also Kellogg, 1994; Kim, 2020); it takes sustained effort and years of practice to master it (Kellogg, 2008, 2018). Due to its cognitively challenging and time-consuming nature, it creates unique motivational challenges for many students (Bruning and Horn, 2000; Boscolo and Gelati, 2019; Camacho et al., 2021). Gaining a better understanding of the interplay between students’ writing performance in relation to their motivational beliefs and emotional reactions to writing can provide needed insights into how writing operates and develops, and thus has the potential to inform writing practices.

While early cognitive models of writing neglected the role of motivation and affect (Hayes and Flower, 1986; Bereiter and Scardamalia, 1987), subsequent models of writing (Hayes, 1996; Graham, 2018) have justly acknowledged the essential role of motivational and affective variables for learners’ task engagement and writing performance. Motivational beliefs are amenable to change and can be stimulated or curbed by external factors (e.g., the social and learning environment) as well as internal influences, which includes the constellation of beliefs writers hold in their long-term memory (for an overview of different beliefs affecting writing see also Graham et al., 2021).

The WWC (writer(s)-within-community) model (Graham, 2018), which provided the theoretical underpinnings for the current investigation, proposed that the contexts (i.e., communities) in which writing is undertaken and learned, as well as the cognitive capabilities, resources, beliefs, and affective reactions of students in these contexts shape and bound students’ writing development. Motivational beliefs (e.g., self-efficacy, motives for writing, evaluations about the value and utility of writing, and judgments about why one is or is not successful) and emotional reactions (such as anxiety or apprehension) influence whether students engage in writing, how much effort they put forth, and the composing actions they undertake. Simultaneously, emotional and affective reactions to writing moderate writers’ use of needed resources from long-term memory (including motivational beliefs) as well as the control and production processes involved in creating text. Further, motivational beliefs and emotions can act as antecedents or consequences to each other. For instance, students who are successful writers within a community are likely to become more efficacious about their writing, making them less anxious about writing. Anxiety in turn can negatively impact the writing process, eroding students’ efficacy as writers. As a result, motivational beliefs and emotional reactions to writing cannot only influence each other, but also the text writers produce.

According to the WWC model, writers’ motivational beliefs as well as their emotional and affective reactions toward writing are influenced by the varying communities in which they learn to write and their experiences as writers over time. Not only writing, but also motivational beliefs and emotional reactions to writing may therefore differ between students with and without MB. Firstly, students with MB may be subjected to different social, cultural, or historical circumstances which shape their writing experiences (e.g., writing is a tool for self-expression in one’s culture and for educating one’s mind in another culture, Graham, 2018). Secondly, many students with MB in countries like Germany are affected by poverty (Stanat and Christensen, 2006), which is likely to influence their literacy experience in general. Thirdly, students with MB in the first generation may have learned to write (at least in part) in communities that differ from those of non-migrant students and have less experience writing in the language of instruction (German in the current study).

As noted earlier, the writing motivational beliefs of focus in the present study was self-efficacy, which may be defined as context-specific capability beliefs regarding task performance (Bandura, 1997). Thus, writing self-efficacy beliefs relate to capability beliefs regarding communication via writing and mastering writing tasks (Klassen, 2002; Pajares, 2003). In contrast to self-concept in writing, self-efficacy is usually assessed at a skill–or task-specific level and must be carefully matched with respective writing assessment, as students may neither feel equally efficacious across different writing tasks (Pajares, 2003), nor across different stages of the writing process (Bruning et al., 2013).

According to social cognitive theory (Bandura, 1986, 1997), self-efficacy plays a vital role in the arousal of student anxiety, i.e., anxiety, which may embody apprehension but also avoidance behavior, is assumed to stem from the confidence with which individuals address (learning) activities. Consequently, it is assumed that individuals only experience anxiety when they feel inefficacious (Bandura, 1997). However, to date, research on self-efficacy and anxiety is limited in the writing domain. A recent review on writing motivation (Camacho et al., 2021) identified 82 studies involving 24 motivational constructs, which were in almost one-half of the cases unclearly defined or not defined at all. Most studies focused on elementary students while middle school (*n* = 14) and high school students (*n* = 7) received less attention. Predominant in this review were studies on self-efficacy (*n* = 37), with very few studies investigating anxiety (*n* = 2). At the high school level (addressed in this study), only one study included measures of anxiety and self-efficacy. Collie et al. (2016) reported a small and negative correlation (*r* = −0.17) between the writing anxiety of boys and their efficacy. They further indicated that positive factors like efficacy were positively correlated to writing-related outcomes, whereas anxiety was negatively related. However, outcome variables did not involve actual writing tasks.

A study by Paul et al. (2021) found that writing efficacy mediated the association between writing anxiety and students’ reported use of revision strategies for high school students who had average scores on measures of achievement goal orientation. Their findings were consistent with the theoretical position that writing anxiety depletes students’ efficacy for writing, which is beneficial for triggering the use of self-regulation strategies in writing. This mediational effect was not found, however, for students who scored either low or high on all achievement goal orientation measures in writing. Further research is needed, particularly with adolescents, as writing becomes increasingly demanding in secondary school, and writing more extensive texts requires adequate planning, revising, and self-regulation strategies (Graham and Harris, 2000).

Existing research has shown that low motivation and debilitating motivational beliefs are common even among more mature writers, particularly among language learners and/ or learners from minority groups. For instance, studies have reported low writing motivation among Hong Kong L2 learners in secondary school (Lee et al., 2018); declining writing motivation among English learners from grades three to eight in the United States (Graham et al., 2021); declining self-efficacy from fifth to ninth grade in English learners in Singapore (Yeung et al., 2011), low writing self-efficacy and apprehensive feelings about writing in English-speaking Hispanic minority students entering high school in the United States (Pajares and Johnson, 1996; Pajares, 2003); and even low writing self-efficacy among high-achieving first-year university L2 learners in England (Busse, 2013).

While studies systematically exploring learner group differences in writing self-efficacy are scarce, fewer look at writing anxiety. It is well known, however, that some students suffer from apprehension unique to written communication which may even cause them to avoid writing courses and prefer occupations that are perceived as requiring little writing (Daly and Miller, 1975). Writing can be particularly anxiety provoking when conducted in a less familiar language, even for rather proficient language learners due to its inherent challenges to the learner’s identity (Horwitz, 2000). When learners express themselves in a language they are less familiar with, they can feel vulnerable and scared of appearing less competent than usual (Noels, 2009; Taylor et al., 2012).

### 2.2. Relationship between writing self-efficacy, writing anxiety, and writing outcomes

It can be assumed that self-efficacy mediates the effect of other influences such as aptitude (Bandura, 1986), and studies have consistently shown positive relationships between self-efficacy and achievement in general (Multon et al., 1991; Brown et al., 2008; Honicke and Broadbent, 2016) as well as writing self-efficacy and writing performance in particular (Klassen, 2002; Pajares, 2003; Camacho et al., 2021). Additionally, regression analyses suggest that self-efficacy is one of the strongest motivational predictors of writing performance (Camacho et al., 2021).

Studies have further shown that writing anxiety is negatively related to the ability to carry out the writing process successfully and to performance on various measures of writing proficiency or skills (for an overview of early studies see Cheng, 2002; see also Richmond and Dickson-Markman, 1985). However, several variables affect the relationship between anxiety and writing performance (Cheng, 2002). Early evidence suggests that anxiety may be detrimental when writing narrative-descriptive topics involving feelings (Faigley et al., 1981), but such relations tend to disappear in argumentative essays where students who are high in apprehension and low in apprehension achieve similar results (Faigley et al., 1981; Madigan et al., 1996). Other studies further supported the contention that the effect of apprehension tends to disappear when self-efficacy was controlled (Pajares et al., 1999; for an overview see Pajares, 2003), providing some support for the argument that anxiety results from a lack of confidence (Bandura, 1997). However, recent data on writing self-efficacy, writing anxiety, and writing performance is notably lacking, especially for high school students where actual measures of writing performance were not administered (i.e., Collie et al., 2016; Paul et al., 2021).

Additional investigations examining the relationships between efficacy, anxiety, and writing performance are necessary because these linkages are not yet fully understood. We provide two examples to illustrate the diverse connections that may exist between efficacy, anxiety, and performance. One, the potential of self-efficacy to reduce the deleterious effects of anxiety (Paul et al., 2021) may not be realized for some students because they overestimate their writing capabilities (Graham and Harris, 1989; Graham et al., 1993). This can occur for a variety of reasons, including misperceptions by students of the demands of writing, inability to accurately assess their own capabilities, or purposefully overestimating capabilities for protective reasons (Bandura and Schunk, 1981). Whatever the cause, an inflated sense of efficacy is not likely powerful enough to fully constrain all of the negative effects of anxiety. Two, while excessive anxiety can inhibit students’ performance on academic tasks (Pekrun and Stephens, 2012), moderate or normal levels of anxiety can be beneficial if it induces greater arousal or an optimal use of cognitive resources (Paul et al., 2021). Consequently, writing anxiety has the potential to enhance students’ writing performance if it is experienced as eustress, even for students who are less confident about their writing capabilities.

### 2.3. The role of students’ migration backgrounds in the relationship between self-efficacy, anxiety, and achievement

Self-efficacy beliefs are strongly influenced by personal accomplishments or mastery experience (Bandura, 1997). Lower levels of self-efficacy are likely to be evident among students with MB, as writing achievements are typically lower among these students, particularly those not speaking the language of instruction at home (for evidence from Germany, see Neumann, 2014, 2017; Müller and Busse, 2023). Yet, self-efficacy is vital for overcoming obstacles when working on challenging tasks (Bandura, 1997). Particular challenges can arise for students with MB from insufficient language fluency which slows down retrieval of content from long-term memory, which is necessary for higher level thinking processes required for writing (Abu-Rabia, 2003; Weigle, 2005; Galbraith, 2009).

It is further likely that the extent to which self-efficacy is related to writing achievement for students with MB can vary as a function of different writing outcomes. In line with this assumption, a recent meta-analysis found the relationship between writing self-efficacy and writing achievement was stronger in L2 (*r* = 0.441) than in L1 (*r* = 0.233) learners (Sun et al., 2021). However, this study mostly focused on self-efficacy for writing when learning English as a foreign language (EFL), and the reported associations may not hold for students with MB or those learning a language other than English because self-beliefs and motivations may well be different for these students (Busse, 2017; Dörnyei and Al-Hoorie, 2017).

In contrast to findings with L2 students, research on academic self-efficacy and academic outcomes with migrant students in the United States have produced mixed results. For instance, a study with Hispanic students in the United States failed to detect a relationship between self-efficacy and grade point average (Niehaus et al., 2011), although self-efficacy was a significant predictor of math achievement and school attendance. In another study with Latino college students, self-efficacy only predicted college performance in second-generation immigrants, not first-generation immigrants (Aguayo et al., 2011). A systematic review of Latino youth in the United States by Manzano-Sanchez et al. (2018) reported that significant relationships are usually not found between self-efficacy and academic attainment for first-generation immigrants.

It is known that there are some cross-cultural differences regarding self-efficacy, including higher instances of self-efficacy in Latinos and lower ones in the self-efficacy of persons of Asian descent (Scholz et al., 2002). A logical extension of these findings is that the relationship between self-efficacy and educational attainment can differ according to cultural background (Brown and Lent, 2006). Even so, mixed results in studies comparing persons with heterogenous language levels may arguably also be linked to different language-levels of first- and second-generation migrants and to the use of different measurement instruments, as more global measures of self-efficacy may not be as useful to capture the relationship between self-efficacy and attainment (Brown and Lent, 2006; Manzano-Sanchez et al., 2018; see also Pajares, 2003).

In literacy research, studies have further reported disjunctions between self-efficacy and performance. For instance, minority students often show higher self-efficacy for reading than their peers but significantly lower achievement (Hornstra et al., 2013; Schöber, 2017). Furthermore, a study with secondary students in Germany revealed that academic self-efficacy did not predict attainment in reading and mathematics in students with MB, as opposed to students without MB (McElvany et al., 2018). Comparable data for writing self-efficacy is unavailable, but mismatches between generally positive writing self-efficacy beliefs and weak writing performance have been observed with low-proficient EFL writers in secondary school (Siekmann et al., 2023) as well as with minority children in primary school (Graham et al., 2005). One may thus assume that students with lower proficiency and/ or students with MB are not always able to assess their capabilities accurately (Graham and Harris, 1989).

Similarly, we could not identify studies on writing anxiety in students with MB. However, it has long been recognized that writing anxiety, particularly fear of making language mistakes, can impact writing achievement in language learners (Horwitz et al., 1986; Cheng, 2002). While decreasing writing anxiety in language learners should lead to better writing performance (Balta, 2018), prior investigations have produced mixed findings regarding the relationship between L2 writing anxiety and L2 writing performance (for an overview of early research see Cheng, 2002). Some studies failed to obtain a significant relationship between writing performance and writing anxiety (e.g., Lee, 2005), whereas writing anxiety positively predicted performance on writing tasks among L2 learners in other investigations (Payant et al., 2019). Contradictory findings could be due to ethnolinguistically diverse samples, but also to the use of different measures to assess anxiety and writing performance. Of particular importance to the present study, Cheng (2004), found negative correlations with the willingness to take writing courses, writing motivation, writing self-efficacy, and writing performance in L2 learners when using a measurement based on three subcomponents of writing anxiety (somatic, cognitive, and avoidance behavior). Her assessments for writing anxiety were employed in the current study.

Regarding the relationship between self-efficacy, anxiety, and achievement, another study with L2 learners (Woodrow, 2011) similarly showed that students with a higher level of self-efficacy tend to have lower writing anxiety levels. Yet, self-efficacy mediated the relation between writing anxiety and writing performance (Paul et al., 2021), which would align with social cognitive theory (Bandura, 1986, 1997) and tie in with non-language learners’ results (Pajares, 2003). However, studies conducted to date concentrate on foreign language learners, whereas research on students with MB is notably missing.

## 3. The present study

The overall aim of the larger research project from which this study was derived was to support less proficient writers in composing full texts. In a previous study, we found that students with MB showed significantly lower achievement when writing in German, both in argumentative and instructional texts (Müller and Busse, 2023). In the current study, we focused our attention on these writing outcomes in German and extended this previous work by examining two motivational variables as key antecedents of writing performance. More specifically, we examined differences between students with and without MB in writing self-efficacy and writing anxiety, and the interplay between these two variables and text quality. Hence, we addressed the following research questions:

RQ1: Are there differences in writing self-efficacy and writing anxiety levels between students with and without migration background?

Findings regarding self-efficacy in students with MB are mixed. However, based on the tenets of the WWC model of writing (Graham, 2018, discussed earlier) and lower competence levels revealed in our previous study (Müller and Busse, 2023), we predicted that students with MB would evidence lower self-efficacy (H1a) in both self-efficacy scales (self-efficacy for establishing *structure and coherence* and self-efficacy for *evaluating and revising*) and higher writing anxiety (H1b) than students without MB.

RQ2: Are writing self-efficacy and writing anxiety predictors of text quality?

First, we examined bivariate relationships and hypothesized a positive relationship between the writing self-efficacy scales and text quality (H2a) and a negative relationship between writing anxiety and text quality (H2b) when the full sample is considered. Second, we investigated multivariate relationships of self-efficacy and anxiety for the full sample as well as their interplay when predicting text quality. We expected that writing self-efficacy would positively predict text quality (H2c), while the relationship between writing anxiety and text quality should disappear when self-efficacy is controlled for (H2d). Our predictions were based on the predicted value of efficacy for enhancing students’ writing, as well as prior research showing that the effect of apprehension tends to disappear when self-efficacy is controlled for (Pajares et al., 1999; see also Pajares, 2003), and that writing efficacy can mediate the effects of anxiety on writing (Woodrow, 2011; Paul et al., 2021). Additionally, we wanted to shed light on the in-depth patterns of the interplay of both self-efficacy and anxiety when predicting text quality in order to explain possible changes in main effects in a multivariate model.

RQ3: Are there differences in the patters of writing self-efficacy and writing anxiety as predictors of text quality for children with and without migration backgrounds?

Finally, we examined multivariate relationships of self-efficacy and writing anxiety as well as their interplay when predicting text quality for students with and without MB separately but did not put forward a hypothesis regarding possible differences. Although stronger relationships between self-efficacy and writing outcomes in L2 learners than in L1 learners have been observed (see the meta-analysis by Sun et al., 2021), studies often fail to detect a relationship between self-efficacy and achievement in students with MB (see the systematic review by Manzano-Sanchez et al., 2018). Writing anxiety is usually negatively related to L2 writing performance (Cheng, 2002) but has also been observed to be a positive predictor of L2 writing (Payant et al., 2019). We did not make any predictions given the general scarcity of studies involving students with MB and writing achievement measures and the inconclusive evidence from existing studies.

## 4. Methods

### 4.1. Design and participants

The study was part of a larger writing feedback intervention project in Germany focusing on adolescents in lower and middle-performance track schools. These schools usually have large numbers of less proficient writers (see also Müller and Busse, 2023). For this article, we examined pre-intervention data and conducted a cross-sectional study drawing on a sample of 208 students in German classes in Year 9 (*M*_age_ = 14.03, SD_age_ 0.75; *n*_girls_ = 91, *n*_boys_ = 112). About half of the sample had migration backgrounds (first- and second- generation; see Table 1 for information on student characteristics), and about half of the sample either speaks German and another language or exclusively other languages than German at home.

**Table 1:** Sample characteristics.

	*N*	%
Gender		
Female	91	44.2
Male	112	54.4
Migration background		
Without	98	48.0
With	106	52.0
Family languages		
German	110	53.1
German and others	67	32.4
Other than German	30	14.5
Place of birth		
Germany	180	81.8
Other than Germany	28	12.7

Due to individual missing data, some subgroups do not add up to the total sample size of *N* = 208.

### 4.2. Procedure and instruments

Data collection took place in early 2020. We assessed writing self-efficacy for establishing *structure and coherence* as well as self-efficacy for *evaluating and revising*. These measures were adapted from a scale by Busse (2013). A writing anxiety scale administered at the same time was adapted from a scale by Cheng (2004). All scales were based on a six-point Likert scale ranging from 1 (not true at all) to 6 (very much true) and showed satisfactory Cronbach’s Alpha above 0.80 for the samples of students in this investigation (see Appendix 1).

Text quality was assessed by analyzing *structure* and *coherence* in an argumentative and an instructional text (*N* = 415 texts) written by students: For the argumentative text, we used an independent writing task from the TOEFL iBT® writing assessment, which was publicly available on the TOEFL website and was used in previous studies to assess students’ writing competence (e.g., Fleckenstein et al., 2020). With this task, students were presented with a statement that they could agree or disagree with: “A teacher’s ability to get along well with students is more important than excellent knowledge of the subject.” When writing their response, students were asked to give reasons to support their opinion. For the instructional text, a prompt from a large-scale study of multilingual language development was administered (MEZ-project, e.g., Klinger et al., 2019), which had been adapted from an instrument developed for writing instructional texts (Reich et al., 2009). With this task, students had to write an article with instructions on how to build a gingerbread house and were provided with nine photographs showing them how to do this. Both of the writing tasks were consistent with curricular expectations for writing in Year 9 in German schools (KMK, 2004). All tasks and instructions were provided in German; texts were assessed according to structure and coherence. This focus draws on findings that structure and coherence are key aspects of text quality (e.g., Plakans and Gebril, 2017). The instrument to assess text structure and coherence in the present study was described in detail in previous works (Siekmann et al., 2022; Müller and Busse, 2023). Students could obtain a theoretical maximum of 17 points of which 8 points were administered for structure (for dividing the text into introduction, main body, and conclusion with relevant content and paragraph breaks) and 9 points were administered for coherence (for consistently referencing a thesis statement throughout the text, for providing ideas supported by appropriate explanations, and for logical connecting words).

### 4.3. Data analyses

This study used an existing sample, but *a posteriori* power analysis set at 90% power, with a 5% significance level, and a conservative small effect size (*f^2^* = 0.15) was conducted to determine minimal samples of students needed for this study. The outcomes of the power analysis revealed a minimum sample size of 70 participants for RQ1, 88 participants for RQ2 and 59 participants for RQ3, which were all smaller than the sample included in this investigation thus indicating sufficient power. All effect sizes will be presented using the standardized regression coefficient β and will be interpreted according to Funder and Ozer (2019), such that an effect between 0.05 < |β| < 0.2 is interpreted as small, an effect between 0.2 ≤ |β| < 0.3 is interpreted as medium, and an effect |β| ≥ 0.3 is interpreted as large. All models were computed using maximum likelihood estimation. Statistical analyses were conducted with R Studio (version 1.1.463; R Core Team, 2018) using the *tidyverse* packages (Wickham et al., 2019) for data management and cleaning. Multilevel mixed-effects models (i.e., multilevel correlations and multilevel *t*-tests) were run using *lme4* package (Bates et al., 2015) and response surface analyses were run using the *RSA* package (Schönbrodt and Humberg, 2021).

To investigate RQ1 with the hypothesis that students with MB would evidence lower self-efficacy (H1a) and higher writing anxiety (H1b) than students without MB, we conducted multilevel *t*-tests with students (level 1) nested in classes (level 2) to determine if there were significant differences in self-efficacy for *evaluating and revising*, in self-efficacy for *establishing structure and coherence* or in writing anxiety between students with and without MB. Means and standard deviations or standard errors will be presented for both groups as well as an effect size as standardized regression coefficient β for each variable.

To answer RQ2 multiple models were applied. First, we hypothesized positive bivariate relationships between text quality and self-efficacy for *evaluating and revising* (H2a), text quality and self-efficacy for *establishing structure and coherence* (H2a), and negative correlations between text quality and writing anxiety (H2b) and presented the full correlation matrix. To examine these bivariate relationships, multilevel bivariate correlations with students (level 1) nested in classes (level 2) were run. Relationships are presented as standardized regression coefficient β.

Second, we investigated the multivariate relationships between text quality, writing self-efficacy and writing anxiety. To do so, we investigated the main effects of self-efficacy and writing anxiety when predicting text quality. Generally, and based on existing literature, we hypothesized positive main effects of self-efficacy (H2c) but no significant main effect of writing anxiety (H2d) in a multivariate model. Then we explored the interplay of both variables to get further insights into these complex relationships. To do so, we run six polynomial regression and response surface analyses computing the interaction model for both self-efficacy scales in the full sample (Models A and D) in students with MB (Models B and E) and in students without MB (Models C and F). Within the interpretation process, the main effect of writing anxiety (regression weight b_1_), the main effect of self-efficacy (b_2_), and the interaction effect (b_4_) will be interpreted. Further, regression weights (a_1_–a_4_) and the shape of the surfaces are considered (Humberg et al., 2019). The line of congruence (LOC) and the line of incongruence (LOIC), whose positions in the coordinate system are determined by the parameters a_1_–a_4_, provide further details about the interplay of self-efficacy and writing anxiety when predicting text quality. These values should be considered together with the Figures illustrating the visual representation of surface for interpretation. Here, a_1_ gives information regarding a potential linear additive effect on the LOC, where positive parameters would indicate that both main effects add up when predicting text quality. The parameter a_2_ indicates if there is curvature on the LOC, which needs to be interpreted together with the plot and shows whether the potential linear effect has a curvature shape or not. The parameter a_3_ shows if the ridge is shifted away from the LOC and gives insight into the shape of the surface. The parameter a_4_ shows if there is curvature on the LOIC, which would indicate that values with large differences between self-efficacy and anxiety lead to differences in text quality. All main and interaction effects are presented as a regression weight b and an effect size in the metric of β, while a_1_-a_4_ are presented in regression weights in the metric of the scales.

**Figure 1:**
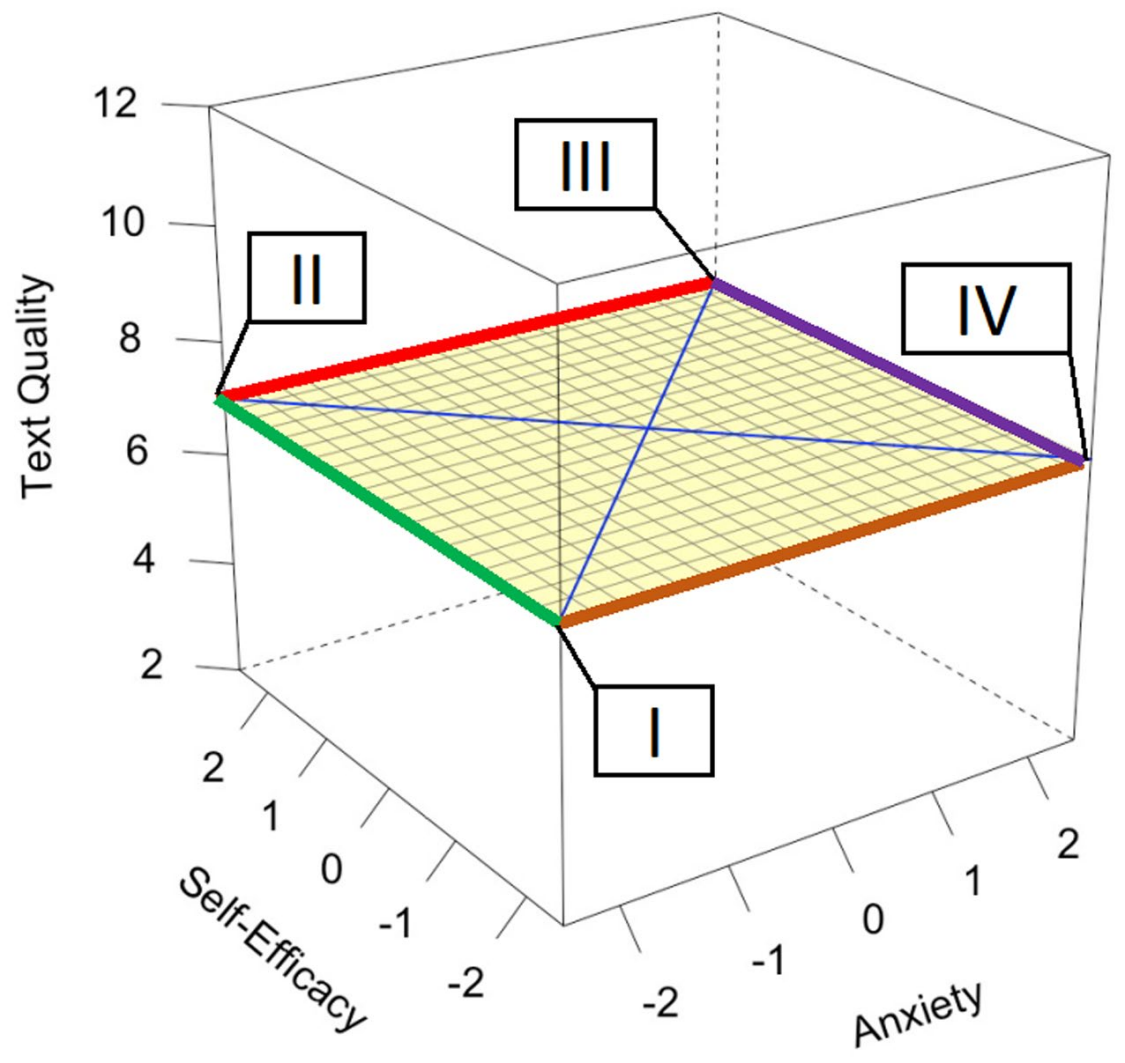
Illustration of the response surface analysis and how it can be interpreted.

Polynomial regression and response surface analysis combine multiple regression with two independent variables to one dependent variable. Typically, the analysis goes along with a comprehensive framework for testing and interpreting the features of resulting three-dimensional graphed relationships. In Figure 1, we present how we interpret the model and how the surface can help. The 3D plot is built by writing anxiety on the x-axis, self-efficacy on the y-axis, and text quality on the z-axis. Writing anxiety and self-efficacy are scaled (grand mean) in the modeling process. In Figure 1, a flat surface is displayed for zero relationship between both predictors and text quality with an intercept of 7. The LOC lies between the points I and III, while the LOIC lies between II and IV. In addition, to better explain the pattern in our data and simplify our results, we used the extremes of self-efficacy and anxiety, which resulted in four groups illustrated by the corners of the surface I-IV: Students with low self-efficacy and low writing anxiety (I), students with high self-efficacy and low writing anxiety (II), students with high self-efficacy and high writing anxiety (III), and students with low self-efficacy and high writing anxiety (IV). Further, the main effects for specific values of the other variable can be illustrated. The green line can be interpreted as the main effect of self-efficacy for low anxiety levels and the purple line for high anxiety levels. The red line can be interpreted as main effect for anxiety, for high self-efficacy, and the brown line for low self-efficacy.

## 5. Results

First, some descriptive values are presented. Students’ scores varied from 0 to 13 points in text quality (*M* = 7.16, SD = 2.04; theoretical maximum = 17 points) and text quality showed an ICC of 0.24, which shows that 24% of the total individual differences in text quality occurred at the class level. Students showed low levels in writing anxiety (range: 1–4.22, *M* = 2.44, SD = 0.90), and moderate levels in self-efficacy for *evaluating and revising* (range: 1.5–6, *M* = 4.49, SD = 0.87) and self-efficacy for *establishing structure and coherence* (range: 1–6, *M* = 4.21, SD = 0.92). To answer RQ1, multilevel *t*-tests were run. Contrary to our prediction for H1a, no significant differences were found for self-efficacy, neither regarding self-efficacy for *evaluating and revising* (with MB: *M* = 4.20, S.E. = 0.10; without MB: *M* = 4.22, S.E. = 0.10; β = 0.02, *p* = 0.831) nor regarding self-efficacy for *establishing structure and coherence* (with MB: *M* = 4.55, S.E. = 0.09; without MB: *M* = 4.43, S.E. = 0.09; β = −0.07, *p* = 0.341). Surprisingly, and contrary to our prediction for H1b, we even found slightly lower levels of writing anxiety among students with MB, differences were significant with a small effect size (with MB: *M* = 2.59, S.E. = 0.10; without MB: *M* = 2.32, S.E. = 0.10; β = 0.15, *p* = 0.044).

To answer RQ2, we first investigated multilevel correlations. Overall, and in line with H2a, text quality was significantly and positively related to self-efficacy, with the data showing a small effect size regarding self-efficacy for *evaluating and revising* and a medium to large effect size regarding self-efficacy for *establishing structure and coherence*. In line with H2b, writing anxiety negatively correlated with text quality, albeit with a small effect size. Writing anxiety was also negatively correlated to both self-efficacy scales with comparably large effect sizes (see Table 2).

**Table 2:** Multilevel correlations between text quality, writing self-efficacy, and writing anxiety.

	1	2	3	4
1. Text quality	1	0.31 (*p* < 0.001)	0.15 (*p* = 0.03)	−0.13 (*p* = 0.029)
2. Writing self-efficacy for *establishing structure and coherence*		1	0.62 (*p* < 0.001)	−0.48 (*p* < 0.001)
3. Writing self-efficacy for e*valuating and revising*			1	−0.42 (*p* < 0.001)
4. Writing anxiety				1

Correlations are multilevel correlations considering class as a nesting factor and are presented in the metric β.

**Figure 2:**
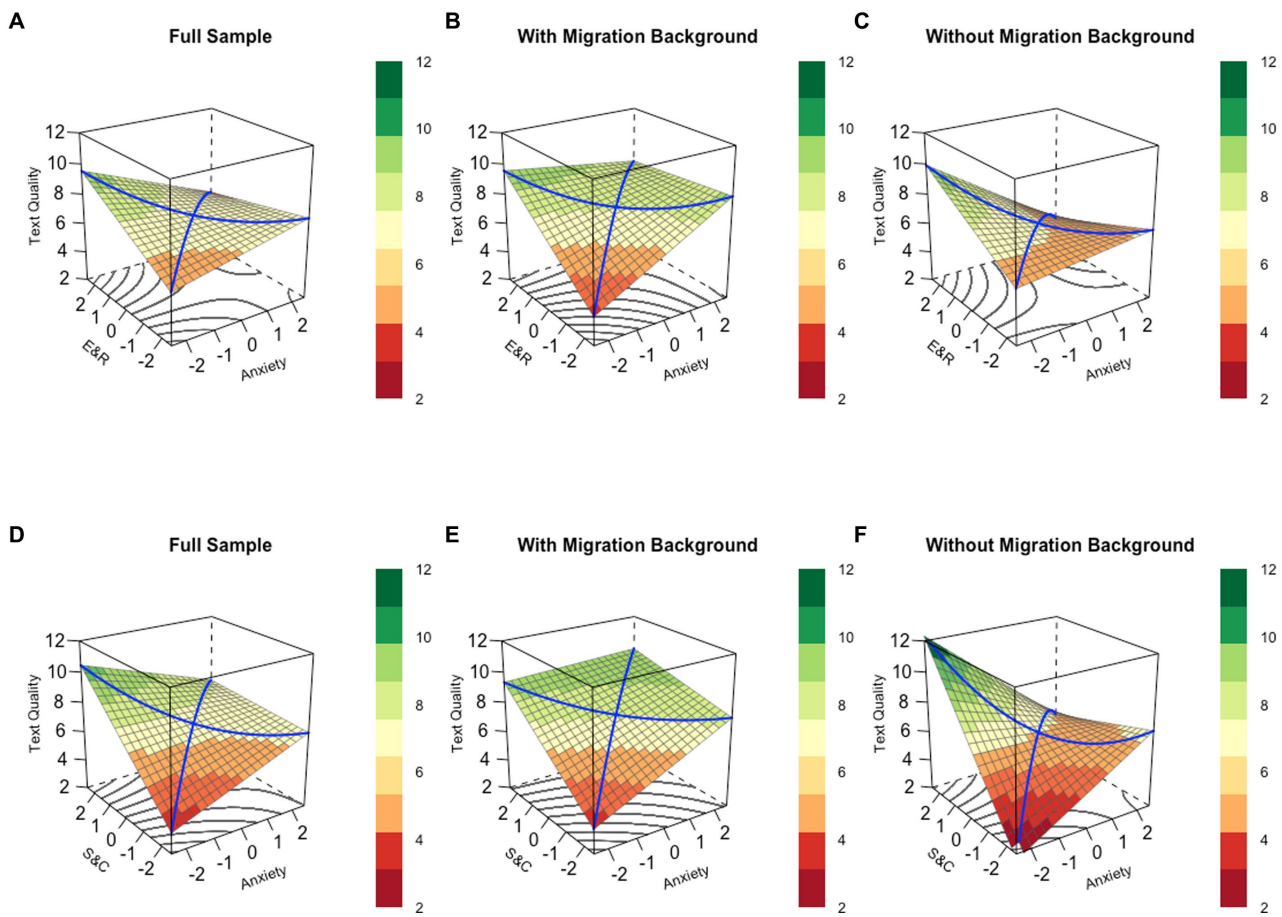
Interaction effects of the scaled writing self-efficacy [**(A–C)** self-efficacy for evaluating and revising, E&R: **(D–F)** self-efficacy for structure and coherence, S&C] and scaled writing anxiety on text quality based on response surface analysis. Values −2 to +2 indicate the range of 95% of the participants. For text quality, values originally ranged from 0 to 13. The surface is the predicted surface that help to interpret the statistical values. The blue lines are the line of congruence from bottom to top and the line of incongruence from left to right. Red color at the surface indicates low text quality, while green color indicates high text quality.

Second, polynomial regression and response surface analyses were run to investigate the interplay of both writing self-efficacy scales and writing anxiety when predicting text quality. Results of the polynomial regression and response surface analyses are presented in Table 3 and Figure 2. In line with H2c, writing self-efficacy for *evaluating and revising* showed a significant main effect in the full sample, but with a small effect size (β_b1_ = 0.132, *p* = 0.04) in Model A, whereas self-efficacy for establishing *structure and coherence* showed a statistically significant main effect with a large effect size (β_b1_ = 0.373, *p* < 0.001) in Model D. We further found that writing anxiety was not significantly related to text quality (main effects in Models A and D). In contrast to the bivariate results and in line with H2d, the main effect of writing anxiety disappeared in the full sample when controlling for self-efficacy.

**Table 3:** Polynomial regression and response surface analyses regarding the interaction of writing self-efficacy (A, B, C: evaluating and revising, D, E, F: structure and coherence) and writing anxiety on text quality.

	*b*	SE	CI lower	CI upper	β	*p*
**Full Sample**						
A: self-efficacy evaluating and revising (*R*^2^ = 0.05)						
Intercept	7.075	0.153	6.776	7.374	3.478	< 0.001
writing anxiety	−0.157	0.154	−0.459	0.145	−0.077	0.154
writing self-efficacy evaluating and revising	0.269	0.154	−0.033	0.572	0.132	0.040
writing self-efficacy evaluating and revising * writing anxiety	0.224	0.114	−0.446	−0.001	−0.114	0.049
D: self-efficacy structure and coherence (*R*^2^ = 0.135)						
Intercept	7.064	0.141	6.787	7.341	3.473	<0.001
writing anxiety	0.067	0.157	−0.241	0.375	0.033	0.353
writing self-efficacy structure and coherence	0.758	0.164	0.436	1.080	0.373	<0.001
writing self-efficacy structure and coherence * writing anxiety	−0.264	0.114	−0.489	−0.040	−0.132	0.021
With migration backgrounds (*N* = 98)						
B: self-efficacy evaluating and revising						
Intercept	7.626	0.199	7.236	8.015	4.325	<0.001
writing anxiety	0.368	0.181	0.012	0.723	0.208	0.043
writing self-efficacy evaluating & revising	0.513	0.194	0.133	0.893	0.289	0.008
writing self-efficacy evaluating & revising * writing anxiety	−0.250	0.171	−0.584	0.085	−0.144	0.144
E: self-efficacy structure & coherence						
Intercept	7.658	0.182	7.301	8.014	4.343	<0.001
writing anxiety	0.479	0.198	0.092	0.867	0.271	0.015
writing self-efficacy structure & coherence	0.751	0.149	0.460	1.043	0.423	0.001
writing self-efficacy structure and coherence * writing anxiety	−0.158	0.119	−0.391	0.074	−0.104	0.182
Without migration background (*N* = 106)						
C: self-efficacy evaluating and revising						
Intercept	6.535	0.223	6.098	6.972	3.056	<0.001
writing anxiety	−0.493	0.271	−1.025	0.039	−0.230	0.069
writing self-efficacy evaluating and revising	0.162	0.238	−0.305	0.629	0.075	0.497
writing self-efficacy evaluating and revising * writing anxiety	−0.279	0.189	−0.649	0.091	−0.138	0.140
F: self-efficacy structure and coherence						
Intercept	6.476	0.196	6.091	6.860	3.029	< 0.001
writing anxiety	−0.193	0.230	−0.643	0.257	−0.090	0.401
writing self-efficacy structure and coherence	0.847	0.236	0.385	1.309	0.395	< 0.001
writing self-efficacy structure and coherence * writing anxiety	−0.518	0.229	−0.966	−0.070	0.191	0.023

*b*, estimate, SE, standard error, CI, Confidence Interval, β, standardized estimate, *p*, p value tested 2-sided.

However, there were statistically significant negative interactions (b_4_) in the two models that both followed a similar pattern that explain why the main effect of writing anxiety disappeared. The shapes of all interaction effects are illustrated in Figures 2A–F. For Model A and self-efficacy for *evaluating and revising*, there was no significant linear additive effect on LOC (*a_1_* = 0.112, *p* = 0.67) but a significant curvature on the LOC (*a_2_* = −0.224, *p* = 0.049); the ridge is shifted away from the LOC (*a_3_* = −0.462, *p* = 0.008) and there is a curvature on the LOIC (*a_4_* = −0.224, *p* = 0.049). For Model D and self-efficacy for establishing *structure and coherence*, there was a significant linear additive effect on LOC (*a_1_* = 0.825, *p* = 0.003) and a significant curvature on the LOC (*a_2_* = −0.264, *p* = 0.021); the ridge is shifted away from the LOC (*a_3_* = −0.691, *p* < 0.001) and there is a curvature on the LOIC (*a_4_* = 0.264, *p* = 0.021). This pattern indicates that the statistically non-significant effect of writing anxiety – in contrast to the bivariate results – can be explained by the fact that students with higher levels of self-efficacy (both scales) showed a negative relationship between anxiety and text quality, whereas students with lower levels of self-efficacy showed a positive relationship between anxiety and text quality.

Third, four polynomial regression and response surface analyses were run to investigate RQ3. When exploring differences between students with and without MB, differentiated effects were found, which are illustrated in-depth using response surface analyses (see Figure 2). In general, for students with MB, writing anxiety had a significant positive small to medium main effect on text quality (see Table 3). This main effect was present in combination with both self-efficacy scales. Additionally, there were positive and statistically significant additive effects for both writing anxiety and writing self-efficacy.

Specifically, for students with MB in Model B for self-efficacy for *evaluating and revising*, there was a statistically significant medium main effect (*b* = 0.513, β = 0.289, *p = 0*.008), and a significant medium main effect of writing anxiety (*b* = 0.368, β = 0.208, *p* = 0.043), but no significant interaction (*b* = −0.250, β = −0.144, *p* = 0.144). Further, there was a linear additive effect (*a_1_* = 0.881, *p* = 0.007), no curvature on the LOC (*a_2_* = −0.250, *p* = 0.144); the ridge was not shifted away from the LOC (*a_3_* = −0.146, *p* = 0.428) and there was no curvature on the LOIC (*a_4_* = 0.250, *p* = 0.144). For Model E, there was a significant large main effect for self-efficacy for *establishing structure and coherence* (*b_2_* = 0.751, β = 0.423, *p* < 0.001), and a medium main effect for writing anxiety (*b_1_* = 0.479, β = 0.271, *p* = 0.015), but no significant interaction (*b_4_* = −0.158, β = −0.104, *p* = 0.182). Moreover, there was a significant linear additive effect (*a_1_* = 1.231, *p* < 0.001), but there was no curvature on the LOC (a_2_ = −0.158, *p* = 0.182); the ridge was not shifted away from the LOC (*a_3_* = −0.272, *p* = 0.117) and there was no curvature on the LOIC (*a_4_* = 0.158, *p* = 0.182). These patterns indicate that self-efficacy (for both scales) and– ounterintuitively–writing anxiety have a positive relation to text quality for students with MB and low self-efficacy levels, which would not have been found in the bivariate relationships alone nor in the full sample. However, the curvature on the LOC for self-efficacy for *evaluating and revising* shows that high levels of self-efficacy and/or anxiety do not change the relationship to text quality (*cf.* flat surface in the back of the cube; Figure 1B). Especially, for students with MB and high self-efficacy, writing anxiety shows no relationship with text quality, but for students with MB and low self-efficacy, higher anxiety relates to better text quality.

In contrast, for students without MB, there was no significant main effect for writing anxiety in both models (*p*s > 0.05). However, in Model F there was a significant and large main effect in self-efficacy for *establishing structure and coherence* (*b* = 0.847, β = 0.395, *p* < 0.001) and a significant interaction (*b* = −0.518, β = 0.191, *p* = 0.023). The interaction effect can be explained by these values: there is no significant additive effect on the LOC (*a_1_* = 0.654, *p* = 0.096), but a significant curvature on the LOC (*a_2_* = −0.518, *p* = 0.023); the ridge was significantly shifted away from the LOC (*a_3_* = −1.040, *p* < 0.001) and there was a significant curvature on the LOIC (*a_4_* = 0.518, *p* = 0.023). In Model C involving self-efficacy for *evaluating and revising*, there were neither statistically significant main effects, nor interaction effects for self-efficacy or writing anxiety (*p*s > 0.05). Further, there were no linear additive effects (*a_1_* = −0.331, *p* = 0.460), no significant curvature on the LOC (*a_2_* = −0.279, *p* = 0.140); the ridge was significantly shifted away from the LOC (*a_3_* = −0.655, *p* = 0.008) and there was no significant curvature on the LOIC (*a_4_* = 0.279, *p* = 0.140). These patterns indicated that self-efficacy (for both scales) and writing anxiety do not add up (i.e., adding up would mean that both main effects are separately important to predict text quality) and show a different pattern in students without MB. However, the curvature on the LOC for self-efficacy for *evaluating and revising* shows that for high levels of self-efficacy and/or anxiety the slope of the LOC falls to the rear (*cf.* bended surface in the back of the cube; Figures 1C,F). To sum up, writing anxiety shows no (Figure 1C) or a slightly positive relation (Figure 1F) to text quality in students without MB and low self-efficacy levels, but for students without MB and high self-efficacy levels, there is a negative relation between writing anxiety and text quality.

## 6. Discussion

The primary aim of this paper was to investigate the interplay between writing self-efficacy, writing anxiety, and text quality, as well as to explore possible differences in the relationships between these variables for students with and without MB. To answer RQ1, we investigated whether there were differences in students’ writing self-efficacy and writing anxiety. To answer RQ2, we first analyzed the bivariate main effects of self-efficacy, writing anxiety, and text quality; second, we examined the multivariate main effects of these variables, and third, we analyzed their interplay with text quality in students with and without migration background. Despite evidence of lower writing attainment in students with MB (e.g., Müller and Busse, 2023), ours is the first study to systematically explore motivational differences in writing between students with and without MB.

Regarding RQ1, we found that students in the full sample felt moderately efficacious about writing, but writing anxiety was generally low. No differences between students with and without MB were observed regarding self-efficacy. Students with MB even had slightly lower writing anxiety levels than those without MB. These findings are worth highlighting as students with MB in our sample had significantly lower writing attainments, as documented in a previous study (Müller and Busse, 2023).

The failure to find significant differences between students with and without migration background’s self-efficacy was unexpected since the self-efficacy measures and writing outcomes were closely matched. Text quality was measured by assessing structure and coherence (see Siekmann et al., 2022; Müller and Busse, 2023), and self-efficacy focused on *establishing structure and coherence* and *evaluating and revising*. These results were inconsistent with prior investigations showing low self-efficacy beliefs among minority students (Pajares and Johnson, 1996) but align with research showing a relative disjuncture between low attainment and high self-beliefs and aspirations by students with MB (Kao and Tienda, 1998; McElvany et al., 2018). One possible explanation could be that students in lower track schools may assign little value to writing or have limited experience with writing which may result in uncalibrated self-perceptions. Results could also point to cross-cultural differences in academic self-efficacy, for instance, a study looking at sources of self-efficacy among students in Germany found that verbal and social persuasion appear to play a more important role than mastery experience in migrant students’ self-efficacy than in non-migrant students (Gebauer et al., 2021). However, in a previous study, we also observed relatively positive self-efficacy beliefs among low-proficient EFL learners, which declined after a feedback intervention (Busse et al., 2020). We hypothesized that feedback might have destroyed students’ illusions of competence (Kruger and Dunning, 1999). In another study, we observed increasing self-efficacy beliefs after a feedback intervention, although writing attainment did not improve (Siekmann et al., 2023). As both studies were conducted with low-proficient EFL learners, one may also assume that less proficient writers generally have difficulties adequately judging their capabilities, which may explain the discordance between self-efficacy and writing performance found in other studies with struggling writers (Graham et al., 2005; Anastasiou and Michail, 2013).

Regarding RQ2, we found medium to large positive correlations between text quality and self-efficacy for *establishing structure and coherence* and small correlations between text quality and self-efficacy for *evaluating and revising* in the full sample. In contrast, writing anxiety negatively correlated with both self-efficacy scales and text quality. Our results successfully replicated earlier research findings confirming that writing self-efficacy is a significant predictor of writing achievement (Klassen, 2002; Pajares, 2003) while stressing the importance of self-efficacy for *establishing structure and coherence* when measuring deep-level text quality. However, the role of anxiety should not be neglected as when investigating RQ3, an interesting interplay between self-efficacy and writing anxiety emerged that differed between students with and without MB. That is, for students without MB, the interaction effect indicates that for higher levels of self-efficacy, higher anxiety is generally associated with lower writing achievement levels. In contrast, lower self-efficacy and higher anxiety levels are associated with higher writing achievement for students with MB. While our data generally seem to support the notion that individuals experience anxiety when they feel inefficacious (Bandura, 1997), our deeper analyses suggest that the relationship between self-efficacy, writing anxiety, and writing performance is complex and varies across individuals.

To better explain the pattern in our data and simplify our results, we used the extremes of self-efficacy and anxiety, which resulted in four groups: Students with low self-efficacy and low writing anxiety, students with high self-efficacy and low writing anxiety, students with high self-efficacy and high writing anxiety, and students with low self-efficacy and high writing anxiety. We further distinguished between students with and without MB. We discovered that the relationship between self-efficacy, writing anxiety, and writing performance differs between these groups. Notably, anxiety had a positive effect on achievement in low-efficacious students with MB. The latter results are consistent with findings by Han and Hiver (2018) showing that EFL students in middle school with moderate to high levels of self-efficacy performed quite successfully on writing tasks despite elevated levels of writing anxiety. These findings may suggest that anxiety is not always harmful if it goes alongside adequate levels of self-efficay. However, in our data, there was no effect on achievement in low-efficacious students without migration background. Similarly, anxiety did not seem to have an effect on performance among high efficacious students with MB. Practically, this suggests that educators and researchers may want to carefully monitor students’ efficacy and anxiety for writing. For example, students who feel anxious about writing and display low self-efficacy, may need greater attention and assistance when writing and learning to write than anxious students who overall feel more efficacious.

In general, results suggest that students are diverse in their motivational and emotional experiences regarding writing. Future research should thus pay more attention to the interplay between writing self-efficacy and anxiety. The latter seems warranted when looking at students with MB, as self-efficacy and writing anxiety are essential variables when exploring achievement differences between students with and without MB, with self-efficacy for *establishing structure and coherence* being the stronger correlate within the self-efficacy measures. Although students with MB in our sample had heterogenous language backgrounds, our findings also tie in with results showing stronger relationships between self-efficacy and writing outcomes in L2 learners than in L1 learners (see the meta-analysis by Sun et al., 2021), and research stressing the importance of paying attention to writing anxiety in language learners (Horwitz, 2000; Cheng, 2002).

Our findings also underline that diverse (linguistic but also cultural) backgrounds may influence the relationship between self-efficacy, anxiety, and writing outcomes, thus extending previous works showing culture-specific differences in self-perceptions and their relation to achievement (e.g., Scholz et al., 2002; Brown and Lent, 2006; Manzano-Sanchez et al., 2018; Ng et al., 2022). Future studies with larger samples may further explore such differences among large migrant groups common in European countries (e.g., students with Turkish backgrounds in Germany who tend to underachieve, also compared to other migrant groups, Stanat and Christensen, 2006).

Although our work provides important insights into the under-researched area of writing motivation, we recognize several limitations in the study reported here. Firstly, this study is only cross-sectional and does not involve random assignment and thus–strictly speaking–does not allow for causal interpretations. Further analyses of data from T2 are necessary to explore the effect of self-efficacy and anxiety on writing development. Moreover, the sample is drawn from students attending middle and lower-track schools. These schools have a less academic focus and tend to have higher percentages of socially disadvantaged students and students with MB. While our study thus provides valuable insights into students at-risk in writing, results may not be generalized. Future studies would have to explore whether the relationship between writing self-efficacy, writing anxiety, and text quality differ when exploring high-achieving students with and without MB. In addition, studies may want to explore these relationships with different outcomes and types of writing and as there may be genre-specific differences (Faigley et al., 1981; Madigan et al., 1996).

Most importantly, migration background is an umbrella term for a diverse student group. Our analyses would have provided more fine-grained results if we had distinguished between first- and second-generation migrants, as achievement results may differ; students in the first generation generally show lower attainment than students in the second generation (OECD, 2010). In addition, students who speak the test language at home usually show better results than students who do not (Stanat and Christensen, 2006). Our analyses showed that text quality was lower in students speaking exclusively another language at home than in students speaking German and another language at home (Müller and Busse, 2023). However, the relatively small percentage of students who exclusively speak another language at home (*n* = 30) would have limited our analyses. Future studies with large samples may explore family language use in more depth and the age of arrival in first-generation students. There could also be differences between students who are genuine L2 writers and those who learned how to write in Germany.

Last but not least, our results and conclusions must be interpreted against the background of the consumerability problem that always occurs when two scales with different meanings and with different scale interpretations are centered in the response surface analysis, and patterns within the interaction are examined. Here, self-efficacy and anxiety were measured using 1 to 6 Likert scales, but social desirability or individual scale interpretation might have led to different scale interpretations (i.e., it is not as accepted to be anxious compared to confident) by the participants when they filled out the questionnaires.

The strength of our study is that self-efficacy measures were closely matched to the writing assessment, which involved two different writing outcomes (instructional and argumentative texts). In addition, we provide insights into writing anxiety, thus addressing the scarcity of research on writing anxiety. While acknowledging the limitations of cross-sectional data, our findings overall seem to indicate that interventions may have to address writing anxiety in students with MB differently. In learners with low self-efficacy who also suffer from writing anxiety, interventions should not primarily aim at reducing writing anxiety and instead focus on increasing self-efficacy first. In students who suffer from writing anxiety but have higher levels of writing self-efficacy, interventions should first aim at reducing writing anxiety.

## 7. Conclusion

In general, our data corroborate findings revealing positive relationships between writing self-efficacy and writing achievement while adding insights into the interplay between writing self-efficacy, writing anxiety, and text quality. Our data suggest that there are motivational differences between students with and without MB. Writing self-efficacy and writing anxiety both seem to play a more important role in text quality when exploring students with MB than when investigating their peers without MB. We suggest that the effect of interventions could be increased if writing self-efficacy and writing anxiety are *a priori* assessed, as interventions could thus be adapted to differing student needs.

## Data availability statement

The datasets presented in this article are not readily available because the authors do not have permission to share data. Requests to access the datasets should be directed to vbusse@uni-muenster.de.

## Ethics statement

The studies involving human participants were reviewed and approved by Ethics Review Board of Department 5 of the University of Koblenz-Landau. Written informed consent to participate in this study was provided by the participants’ legal guardian/next of kin.

## Author contributions

VB conceived and supervised the overall project and wrote the first draft of the manuscript for this study. SG and TU contributed to the conceptualization of the study and to writing. NM executed the project, collected the data, organized the database and contributed to writing. VB and SG contributed to manuscript revision. TU conducted the statistical analyses and wrote the results section. All authors listed have made a substantial, direct, and intellectual contribution to the work and approved it for publication.

## Funding

This research was funded by the Mercator foundation.

## Conflict of interest

The authors declare that the research was conducted in the absence of any commercial or financial relationships that could be construed as a potential conflict of interest.

## Publisher’s note

All claims expressed in this article are solely those of the authors and do not necessarily represent those of their affiliated organizations, or those of the publisher, the editors and the reviewers. Any product that may be evaluated in this article, or claim that may be made by its manufacturer, is not guaranteed or endorsed by the publisher.

## Figures and Tables

**Table:** 

Scale (number of items)	Items	Reliability (Cronbach’s *α*)
Self-efficacy for *establishing structure and coherence* (4)	This is how I rate myself in terms of writing texts in German… *[So schätze ich mich ein in Bezug auf das Schreiben von Texten auf Deutsch…]*	0.886
	… I can write a good text in German *[…ich kann einen guten Text auf Deutsch verfassen.]*	
	… I can write a well-structured text in German. *[…ich kann einen gut strukturierten Text auf Deutsch verfassen.]*	
	… I can connect my ideas in a meaningful way. *[…ich kann meine Ideen sinnvoll verknüpfen.]*	
	… my German texts are comprehensible for the readers. *[…meine deutschen Texte sind nachvollziehbar für die Leser.]*	
Self-efficacy for *evaluating and revising* (4)	When I write a text in German, I am able to … *[Wenn ich einen Text auf Deutsch geschrieben habe, kann ich …]*	0.844
	… assess the strengths and weaknesses of my text well. *[.. die Stärken und Schwächen meines Textes gut einschätzen.]*	
	… revise the text on my own. *[… den Text selbständig überarbeiten.]*	
	… assess whether or not my text meets the requirements of Year 9 *[… einschätzen, ob mein Text den Anforderungen der 9. Klasse entspricht oder nicht.]*	
	… assess whether or not I have met the writing goals *[… einschätzen, ob ich die Schreibziele erreicht habe oder nicht.]*	
Writing anxiety (9)	This is how I feel when writing in German … *[So fühle ich mich beim Schreiben auf Deutsch …]*	0.821
Somatic (3)	… My mind often goes blank when I write in German. *[…ich habe of einen Blackout, wenn ich auf Deutsch schreibe.]*	
	… I tremble or perspire when I write in German *[…ich zittere oder schwitze, wenn ich auf Deutsch schreibe.]*	
	… I feel my whole body rigid and tense when I write in German. *[…ich fühle mich verkrampft, wenn ich auf Deutsch schreibe.]*	
Cognitive (3)	… If my German composition is to be evaluated, I would worry about getting a poor grade. *[…wenn ich weiß, dass mein Deutschaufsatz benotet wird, habe ich Angst vor einer schlechten Note.]*	
	… I’m afraid of my German composition being chosen as a sample for discussion in class. *[…wenn mein Deutschaufsatz ausgewählt wird, um ihn beispielhaft im Unterricht zu besprechen, habe ich Angst.]*	
	… while writing German compositions, I feel worried and uneasy if I know they will be evaluated. *[…wenn ich weiß, dass mein Deutschaufsatz von Lehrer/innen gelesen wird, bin ich nervös.]*	
Avoidance behavior (3)	… Unless I have no choice, I would not use German to write texts. *[…wenn ich die Wahl hätte, würde ich nicht auf Deutsch schreiben.]*	
	… I avoid writing longer texts in German *[…ich vermeide es, längere Texte auf Deutsch zu schreiben.]*	
	… I usually do my best to avoid writing texts in German *[…wenn es geht, vermeide ich es, auf Deutsch zu schreiben.]*	

## Appendix 1

Overview of scales on perceptions of process-oriented writing and feedback practices.
